# Caregiver strain on informal caregivers when providing care for older patients undergoing major abdominal surgery: a longitudinal prospective cohort study

**DOI:** 10.1186/s12877-020-01579-8

**Published:** 2020-05-19

**Authors:** T. L. Janssen, P. Lodder, J. de Vries, C. C. H. A. van Hoof – de Lepper, P. D. Gobardhan, G. H. Ho, L. van der Laan

**Affiliations:** 1 Department of Surgery, Amphia Hospital, P.O. Box 90518, 4800 RK Breda, The Netherlands; 2 Department of Methodology and Statistics, Tilburg University, Tilburg, The Netherlands; 3 Department of Medical and Clinical Psychology, Tilburg University, Tilburg, The Netherlands; 4 Department of Medical Psychology, Elisabeth-TweeSteden hospital, Tilburg, The Netherlands; 5 Department of Cardiovascular Science, UZ Leuven, Leuven, Belgium

**Keywords:** Informal caregiving, Caregiver burden, Psychological health, Elective surgery, Older patients

## Abstract

**Background:**

Health-care systems nowadays rely on complementary patient care by informal caregivers. The need for, and burden on, informal caregivers will likely increase in the upcoming years. This study aimed to examine the burden on caregivers when providing care for elderly patients undergoing major abdominal surgery.

**Methods:**

A single-centre longitudinal cohort study was conducted between November 2015 and June 2018 in the Amphia hospital in Breda, the Netherlands. Patients aged 70+ undergoing elective surgery for colorectal carcinoma (CRC) or an abdominal aortic aneurysm (AAA) were included in this study. Informal caregiver burden was assessed and compared over time using the Caregiver Strain Index (CSI) at the outpatient clinic visit, at discharge, 2 weeks post-discharge and after 6 and 12 months. The effects of patient- and caregiver-related factors on the experienced caregiver strain were examined.

**Results:**

CSI scores of 248 caregivers were significantly increased at discharge (3.5 vs 2.6; *p* < 0.001) and 2 weeks post-discharge (3.3 vs 2.6; p < 0.001). After 12 months, scores dropped below baseline scores (1.8 vs 2.6; *p* = 0.012). The highest strain was observed 2 weeks post-discharge for AAA patients and at discharge for CRC patients. Older age, physical or cognitive impairment and burden of comorbidity were associated with an increased caregiver strain at baseline. Type of surgery was independently associated with the change in mean CSI scores over time; a bigger change in caregiver burden is observed after open surgery.

**Conclusion:**

In the early postoperative period, perceived caregiver strain was significantly increased. Psychological support for caregivers may be advisable, with timing of this support depending on diagnosis and patient-related factors.

**Trial registration:**

This manuscript was retrospectively registered on 05-04-2016 in the Netherlands Trial Register (NTR5932). http://www.trialregister.nl/trialreg/admin/rctview.asp?TC=5932

## Background

Two diseases that are commonly present in older patients are colorectal cancer (CRC) and abdominal aortic aneurysms (AAA). Both conditions require major abdominal surgery and may both have serious impact on a patient’s physical and mental well-being, thereby possibly requiring additional help of informal caregivers.

In CRC patients, an additional mental burden is experienced due to the diagnosis of cancer, with possible psychological problems and decreased quality of life as a consequence [1, 2]. The quality of life in cancer patients is highly and significantly correlated with their informal caregivers’ quality of life [3]. According to a 2013 Dutch report, 34% of informal caregivers experience overload when providing care for patients who are suffering from psychological problems, compared to 19% of the caregivers of psychologically healthy patients [4]. Providing informal care to cancer patients negatively affects psychological health in over 90% of caregivers and physical health in nearly 10% [5, 6].

Open or endovascular (minimally invasive) surgical repair of an AAA also has a negative effect on the short-term quality of life and health status of the patient [7, 8]. Only one previous study described the impact of AAA repair on informal caregivers [9], even though AAA repair is considered major abdominal surgery and has considerable impact on elderly patients and their caregivers.

The number of elderly people diagnosed with above-mentioned diseases is increasing. Due to population ageing, fewer working people have to pay for the costs of sufficient healthcare for the growing elderly population. To reduce these costs, a shift from institutional to informal or family care is inescapable [10]. Most health-care systems, therefore, rely on informal caregivers to play a significant role by providing complementary care [10].

In the Netherlands, one in three adults (nearly 4.5 million) are informal caregiver and provide any form of short- or long-term care for a spouse, parent, relative, friend, or neighbour [11]. Thanks to these caregivers, both patients’ quality of life and participation in society increases and the burden on and costs of the health-care system decreases [11]. A near 8 billion euro is saved, which otherwise had to be spend on home care services [4].

It is expected that the number of available informal caregivers per 85-year old person will decrease from 30 in 1975 and 15 in 2015, to 6 in 2040 [12]. The demand for and burden on informal caregivers is expected to increase even further due to the aging of the population, the socialisation and extramuralisation of care and budgetary restrictions in professional care [4]. In the Netherlands in 2017, nearly 10% of these caregivers experience the providing of care as a strain and a serious burden [11], influencing caregivers’ quality of life.

In accordance with the Dutch Work and Care act, caregivers can take a 2-week paid leave to provide care for a family member. After these 2 weeks, caregiving must be combined with a regular job. Five in six caregivers between 18 and 65 years old combine informal caregiving with a regular job [11].

The well-being of informal caregivers depends on the interplay between stressors (cognitive impairment, functional disability or problem behaviour of a patient), the number of hours of informal caregiving and mediators (formal services, quality of the relationship, emotional support). An imbalance can lead to overload of the caregiver and decreased well-being [13]. The combination of informal caregiving with full-time employment also increases the risk for overload [14]. In the Netherlands, a little over half of the labour force works full-time.

The aim of this study was to provide an overview of the subjective caregiver strain as experienced by informal caregivers and to examine possible patient- or caregiver-related factors influencing strain on these informal caregivers when providing informal care for elderly patients undergoing major abdominal surgery for CRC or an AAA.

## Methods

### Study design, setting and participants

A single-centre longitudinal cohort study was conducted in the Amphia hospital, a large teaching hospital in Breda, the Netherlands. Patients aged 70 years or older undergoing elective surgery for CRC or an AAA between November 2015 and June 2018 were included in this study. Patients were excluded if they were acutely admitted, needed acute surgery, underwent surgery in the 6 months prior to the first outpatient clinic visit, and when surgery was planned within 2 weeks of the outpatient clinic visit. Patients’ physical and nutritional health status, factors of frailty and haemoglobin levels were optimized by prehabilitation in the five weeks prior to surgery. Informal caregivers were asked to visit the outpatient clinic together with the patient at each time point, to assess the burden experienced by these caregivers when providing care of elderly patients undergoing major abdominal surgery. No specific supporting programmes for informal caregivers were provided by the hospital, however special attention was provided to these caregivers during the first outpatient clinic visit in order to help them prepare for the burden they may experience after discharge. Written informed consent was obtained during trial enrolment, before the first outpatient clinic visit. The prehabilitation protocol has previously been published [15].

### Data collection: informal caregiver characteristics

Informal caregiving was defined as providing any short- or long-term care to a person in the social network in need of care, complementary to institutional care.

Informal caregiver burden was assessed using the Caregiver Strain Index (CSI) [16]. The CSI is a brief and reliable, 13-item dichotomous (yes/no) questionnaire developed by Robinson et al. in 1983 and validated by Post et al. in 2007 [17], designed to measure perceived burden of caregivers when providing care for a patient. The CSI questionnaire in the current sample had a good estimated reliability (Cronbach’s alpha = 0.832). It comprises important domains (employment, financial, physical, social and time) and focusses on stressors which can burden the caregiver when providing care to a patient. The questionnaire has a maximum score of 13 points. A score ≥ 7 represents high strain. Additionally, relation between caregiver and patient, age of caregiver and distance between caregivers’ residence and patients’ residence were registered.

Informal caregivers were asked to complete the CSI during the first outpatient clinic visit (T1), at discharge (T2), 2 weeks post-discharge (T3), after 6 months (T4) and after 12 months (T5). When a patient died, the informal caregiver was no longer asked to complete the CSI.

### Data collection: patient characteristics

The following baseline demographic patient information was assessed: age, gender, cognitive impairment, burden of comorbidities (Charlson Comorbidity Index (CCI)) [18], dependence in activities of daily living (the KATZ-Activities of Daily Living (KATZ-ADL) score) [19], and nutritional health status (Short Nutritional Assessment Questionnaire (SNAQ)) [20]. Patients were considered dependent in ADL when the KATZ score was less than six. Patients with a SNAQ score ≥ 3 were considered malnourished. The discharge location (home or in an institution) was registered.

### Aims

The primary aim was to assess caregiver strain across different time points using the CSI and compare scores over time. Secondary aims were to examine the influence of: 1) patients’ age, 2) dependency in ADL, 3) type of surgery, 4) burden of comorbidity, 5) cognitive impairment and 6) development of delirium on the perceived caregiver strain.

### Statistical analysis

Dichotomous variables were presented as frequencies with percentages. Continuous variables were tested for normality using the Shapiro-Wilk test and presented as medians with interquartile ranges in case of a skewed distribution. Between-group differences for continuous variables were tested for statistical significance using the Mann-Whitney U test. A *p*-value below 0.05 was considered statistically significant. Missing data for the covariates was infrequent and was not imputed.

Linear mixed modelling was performed to examine the differences in mean CSI score between each time point and baseline for each diagnosis and for the total group. Generalized linear mixed models were used for the dichotomized outcome of having a CSI score of seven or higher. An unstructured covariance matrix was used to model the residual (co)variances of the repeated measurements. Analyses have been adjusted for the following covariates: age, cognitive impairment, dependence in ADL, Charlson Comorbidity Index and type of surgery. Test for differences between the mean CSI score at baseline and each of the CSI time points during follow-up were derived directly from the mixed model by making use of the custom hypothesis test command in the SPSS syntax. Because the baseline score was compared to scores at four separate time points, the statistical significance for these tests was set at *p* = 0.0125 (*p* = 0.05 / 4 tests) in order to correct for multiple comparisons. Missing data for the CSI scores was handled through full information maximum likelihood estimation, incorporated in the linear mixed modelling analysis.

For the primary outcome measures, Cohen’s d effect sizes were computed. An effect size between 0.00 and 0.30 was considered small, between 0.30 and 0.60 was considered medium and above 0.60 was considered a large effect.

All data were prospectively stored using the electronic patient file ‘Hyperspace Version IU4 (Epic, Inc., Verona, WI)’ of Amphia Hospital Breda, the Netherlands. Statistical analysis was performed using IBM SPSS statistics software version 24.0 (SPSS Inc., Chicago, Illinois, USA).

This research project has been retrospectively registered in the Netherlands Trial Register (NTR5932).

## Results

**Fig. 1:**
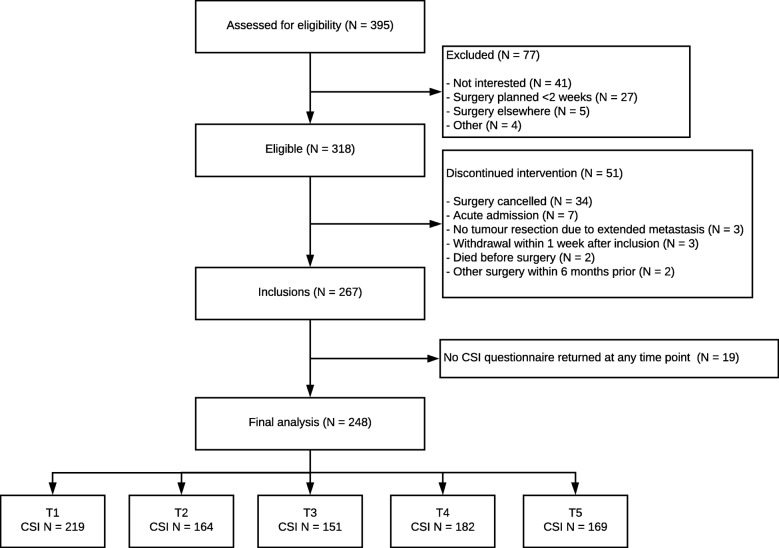
Flow Diagram

A flow diagram from eligibility assessment to inclusion to final analysis is shown in Fig. 1. Eligibility was assessed in 395 patients. A total of 267 patients underwent elective surgery for CRC or an AAA from November 2015 to June 2018 and were included in this study. Of these, 248 patients (93%) had an informal caregiver who filled in the CSI questionnaire at any time point and were therefore included in this analysis. The remaining 19 patients (7%) responded that they did not have an informal caregiver and returned the questionnaires empty.

Sixty-four patients (26%) underwent AAA repair and a colorectal tumour was resected in 184 patients (74%). A complete overview of baseline patient characteristics is presented in Table 1.

**Table 1:** Baseline characteristics of all patients

	AAA ***N*** = 64 (26%)	Colorectal cancer ***N*** = 184 (74%)	Full sample ***N*** = 248 (100%)
Age, median (IQR)	77 (72–81)	77 (74–82)	77 (73–81)
Male gender	53 (83)	108 (59)	161 (65)
**Comorbidities**			
Charlson Comorbidity Index, median (IQR)	6 (4–7)	6 (5–7)	6 (5–7)
Cognitive impairment	1 (1.6)	17 (9.2)	18 (7.3)
**Dependent in ADL/Nutritional impairment**			
KATZ-ADL score ≤ 5	13 (20)	44 (24)	57 (23)
SNAQ score ≥ 3	3 (4.7)	38 (21)	41 (17)
**Type of surgery**			
Open	14 (22)	22 (12)	36 (15)
Minimally invasive	50 (78)	162 (88)	212 (86)
Discharged home	56 (89)	154 (88)	210 (88)

The median age of all caregivers was 70 years old (IQR 54–75), as presented in Table 2. The majority of informal caregivers provided informal care for their spouse (57%) or for a parent (34%). In this cohort, children were more often involved when a patient was suffering from CRC (36% vs 27%). Less than one in seven patients lived over five km away from the patient and only 11 of 219 caregivers (5%) lived over 20 km away.

**Table 2:** Baseline demographic variables of informal caregivers

	AAA ***N*** = 64 (26%)	Colorectal cancer ***N*** = 184 (74%)	Full sample ***N*** = 248 (100%)
Age, median (IQR)	70 (56–74)	70 (53–76)	70 (54–75)
**Relation to patient**			
Children	17 (27)	67 (36)	84 (34)
Spouse	42 (66)	100 (54)	142 (57)
Other relative	2 (3.1)	7 (3.8)	9 (3.6)
Friend/Neighbour	0 (0)	4 (2.2)	4 (1.6)
Unknown	3 (4.7)	6 (3.3)	9 (3.6)
**Distance to patient**			
Living in	41 (64)	104 (57)	145 (59)
0–5 km	14 (22)	46 (25)	60 (24)
Over 5 km	5 (7.8)	27 (15)	32 (13)
Unknown	4 (6.3)	7 (3.8)	11 (4.4)

Table 3 presents mean CSI scores and the number of informal caregivers with a high burden at T1 to T5. A statistically significant increase was observed in overall CSI score in the early postoperative period (T2 3.5 vs 2.6, *p* < 0.001; Cohen’s d 0.339; and T3 3.3 vs 2.6, *p* < 0.001; Cohen’s d 0.269). The highest strain was experienced at discharge. After that, at 6 and 12 months post-surgery, CSI scores dropped below the scores at the outpatient clinic visit. This drop reached statistical significance at 12 months (1.8 vs 2.6, *p* = 0.012; Cohen’s d 0.283), showing a relief of the burden for the informal caregiver once a patient has been treated for his disease. A similar course is seen for the number of patients experiencing a high strain, according to a CSI score ≥ 7. For AAA patients, the highest perceived strain was observed 2 weeks after discharge and was significantly higher compared to baseline (3.7 vs 2.1, *p* = 0.004; Cohen’s d 0.514), while in CRC patients the highest strain, also significantly higher compared to baseline, was observed at discharge (3.6 vs 2.7, *p* = 0.001; Cohen’s d 0.365). In CRC patients, caregiver strain also dropped below baseline after 12 months (1.8 vs 2.7, *p* = 0.011; Cohen’s d 0.306).

**Table 3:** Caregiver strain index per time point for all patients and per diagnosis

	Outpatient clinic visit (T1)	Discharge (T2)	Two weeks post-discharge (T3)	6 months post-surgery (T4)	12 months post-surgery (T5)
**AAA patients**	***N*** **= 54**	***N*** **= 47**	***N*** **= 39**	***N*** **= 46**	***N*** **= 45**
CSI, mean (SD)	2.1 (2.0)	3.1 (3.0)^a, d^	3.7 (3.4)^a, d^	1.4 (1.9)^d^	1.7 (2.6)^c^
CSI Score ≥ 7^b^	3 (5.6)	6 (13)	11 (28)	1 (2.2)	3 (6.7)
**CRC patients**	***N*** **= 165**	***N*** **= 117**	***N*** **= 112**	***N*** **= 136**	***N*** **= 124**
CSI, mean (SD)	2.7 (3.0)	3.6 (3.4)^a, d^	3.1 (3.2)^a, c^	2.3 (2.9)^c^	1.8 (2.7)^a, d^
CSI Score ≥ 7	18 (11)	29 (25)^a^	15 (13)	16 (12)	11 (8.9)
**All patients**	***N*** **= 219**	***N*** **= 164**	***N*** **= 151**	***N*** **= 182**	***N*** **= 169**
CSI, mean (SD)	2.6 (2.8)	3.5 (3.3)^a, d^	3.3 (3.2)^a, c^	2.1 (2.7)^c^	1.8 (2.7)^a, c^
CSI Score ≥ 7	21 (9.6)	35 (21)^a^	26 (17) ^a^	17 (9.3)	14 (8.3)

^a^Significant difference in patient group between time point and outpatient clinic visit (T1; *p* < 0.0125)

^b^Linear mixed modelling not possible due to the low number of events

^c^Small Cohen’s d effect size(< 0.30)

^d^Medium Cohen’s d effect size(0.30–0.60)

**Fig. 2:**
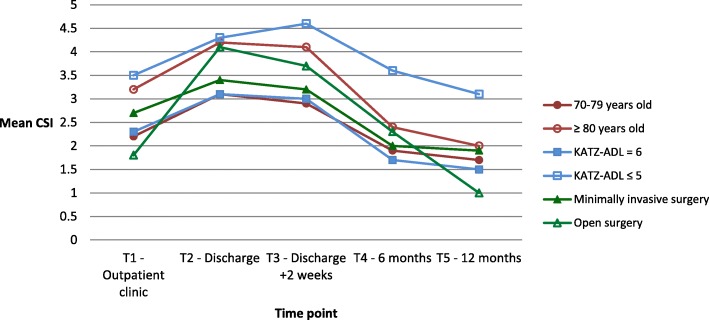
CSI in relation to patients’ age and dependency in ADL and type of surgery

**Fig. 3:**
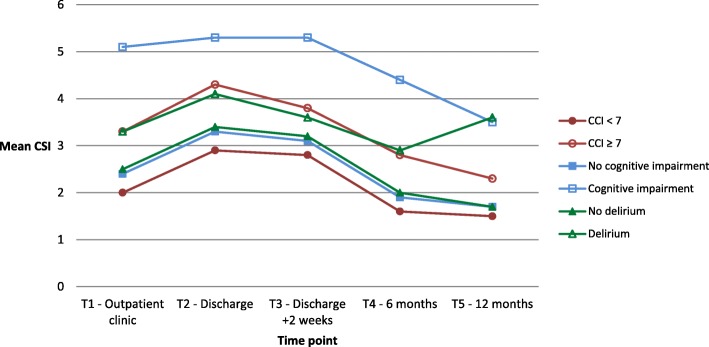
CSI in relation to patients’ burden of comorbidity, cognitive status and development of delirium

The patient- and caregiver-related factors that may influence the experienced informal caregiver strain for caregivers of all patients are presented in Figs. 2 and 3. Tables for these figures, together with tables divided per diagnosis, are added as supplementary material (Appendices A to F).

The interaction between T-moment and type of surgery (F(4,189.615) = 2.594; *p* = 0.038) reached statistical significance, suggesting that type of surgery affects the change in mean CSI score over time. In contrast, the interactions between T-moment and age group (F(4,178.928) = 1.676; *p* = 0.16), dependency in ADL (F(4,170.941) = 1.338; *p* = 0.26), burden of comorbidity (F(4,185.487) = 0.148; *p* = 0.96), cognitive impairment (F(4,171.956) = 0.406; *p* = 0.80) and delirium (F(4,202.095) = 1.165; *p* = 0.33) failed to reach statistical significance.

Figure 2 and appendices A to C present the CSI divided by patients’ age group and dependency in ADL, and type of surgery. Overall, caregivers of octogenarians (80+) had a higher mean CSI score for all time points compared to those providing care for non-octogenarians, however this difference was only statistically significance at baseline (*p* = 0.016). After 6 and 12 months, mean CSI scores for caregivers of octogenarians decreased significantly compared to baseline (*p* = 0.008 and *p* = 0.002 respectively). For caregivers of 70–79 year olds, the experienced strain was significantly increased at T2 and T3 (*p* < 0.001 for both time points). This difference was no longer present after 6 and 12 months. In CRC patients specifically, the mean CSI was significantly higher in caregivers of octogenarians (80+) at T1 to T3 compared to caregivers of 70–79-year olds (*p* = 0.001, *p* = 0.004 and *p* = 0.050 respectively), but these differences were no longer significant after 6 and 12 months.

No significant differences were observed between follow-up and baseline in caregivers of patients that were dependent in ADL. Compared to caregivers of independent patients, caregivers of dependent patients perceived more strain at baseline (*p* = 0.011), after 2 weeks (*p* = 0.035), 6 months (*p* = 0.001) and 12 months (*p* = 0.001). Caregivers of independent patients had significantly higher mean CSI scores at discharge and after 2 weeks compared to baseline (3.2 and 2.9 vs 2.3; *p* < 0.001 and *p* = 0.001 respectively), and a significantly lower mean CSI score after 12 months (1.5 vs 2.3; *p* = 0.004). After 12 months, caregivers of dependent AAA patients perceived more strain (2.9 vs 1.4; *p* = 0.041).

For caregivers of patients undergoing open AAA repair, a statistically significant increase was seen in mean CSI score at discharge (5.2 vs 1.7; *p* = 0.001) compared to baseline. Scores at discharge and after 2 weeks were also significantly higher compared to the endovascular repair group (5.2 vs 2.6, *p* = 0.049; and 6.6 vs 2.9, *p* = 0.008 respectively). For all caregivers, the mean CSI scores were significantly increased at discharge and 2 weeks post-discharge compared to baseline after minimally invasive surgery (*p* = 0.002 and p = 0.001 respectively) and at discharge compared to baseline after open surgery (p = 0.002) No significant differences were observed between groups.

The CSI in relation to patients’ burden comorbidity and cognitive status, and to the development of delirium is presented in Fig. 3 and Appendices D to F. The perceived caregiver strain was significantly higher at baseline and at discharge in caregivers of patients who had a high burden of comorbidity compared to those taking care of patients with a low burden of comorbidity (*p* = 0.001 and *p* = 0.005 respectively). Two weeks after discharge, these differences were no longer present. The perceived caregiver burden was significantly higher at discharge and at 2 weeks after discharge compared to baseline in caregivers of patients with a CCI < 7 (*p* = 0.001 for both time points), and at discharge compared to baseline in caregivers of patients with a CCI ≥ 7 (*p* = 0.010).

Overall, cognitive impairment seemed to influence the perceived caregiver burden, resulting in significantly higher mean CSI scores at baseline (*p* = 0.003), at discharge (*p* = 0.042) and after 6 months (*p* = 0.015) compared to caregivers of cognitively unimpaired patients. Cognitive impairment did not affect differences in mean CSI score between follow-up and baseline. Caregivers of cognitively unimpaired patients perceived a higher burden at discharge and 2 weeks post-discharge compared to baseline (*p* < 0.001 for both time points). No mean CSI scores or differences could be calculated for caregivers of cognitively impaired AAA patients separately, due to a low number of events.

Similar to cognitive status, no mean CSI scores or differences could be calculated for caregivers of AAA patients with and without delirium separately. Caregivers of patients that did and did not develop a delirium during admission had a significantly higher CSI score at discharge and 2 weeks after discharge compared to baseline (*p* < 0.001 for all time points). Caregivers of patients who did not develop a delirium perceived a significantly lower burden after 12 months (*p* = 0.003), while caregivers of patients with delirium had scores that were similar to baseline.

When performing a sensitivity analysis, caregivers who were lost to follow-up provided care for patients who were more often dependent in ADL (*p* = 0.008). No differences were observed in all other baseline variables.

## Discussion

The burden on the health-care system will increase due to population aging and the subsequent increase in the number of elderly patients who require additional care. To relieve this burden, these systems rely on informal caregivers to provide complementary care. Due to developments in society, the demand for and burden on informal caregivers are likely to increase. This may lead to overload and decreased quality of life of these caregivers. This study aimed to describe the caregiver strain experienced by informal caregivers when providing care for elderly patients undergoing elective major abdominal surgery for CRC and AAA and the factors influencing this strain.

Caregiver strain was highest at discharge and 2 weeks post-discharge, emphasizing the importance of informing informal caregivers prior to surgery to prepare them for the care situation at home and of adequate psychological support for both patient and the patients’ informal caregiver during the early postoperative period when desired. Although more research on the clinical significance of differences on the CSI scale is needed, the Cohen’s d effect size for all CSI scores at discharge was medium, suggesting that these changes over time may be clinically relevant. This finding is in line with previous studies on caregiver strain on spouses of patients with laryngeal cancer and caregiver strain following orthopaedic surgery, where the highest caregiver strain is observed in caregivers shortly after discharge [21–23]. However, two of these studies did not assess caregiver strain prior to admission. In the study by Zadzilka et al., in line with the current study, caregiver strain dropped below preoperative scores after 1 year [23]. In another study assessing strain in caregivers of paediatric surgical patients, a decreased score was seen at 3 months post-surgery when compared to baseline [24]. However, the overall caregiver strain was higher in this study and all caregivers experienced a high strain (CSI ≥ 7) for the entire study period, most likely due to the paediatric nature of the study.

After 12 months, the experienced caregiver strain was lower than at baseline, suggesting that surgery was not only effective in treating the patient, but also in lowering overall caregiver burden.

Compared to previous studies, our study on average showed lower caregiver strain on informal caregivers who provide care for elderly patients with cancer [25–28]. Studies using the CSI to assess caregiver strain for caregivers of patients with neurologic diseases or neurodegenerative disorders also demonstrate a higher caregiver strain [29, 30]. However, none of the above-mentioned studies assessed differences in caregiver strain across several time points. Also, no previous study has investigated the effect of abdominal surgery on the caregiver strain over time. In the current study, no more than a quarter of caregivers experience a high strain when providing care for a patient at any time point.

The strain perceived by caregivers of CRC and AAA patients on average differs less than one point on the 13-item questionnaire. This may lead to the conclusion that the burden of these diseases, their treatments and their impact on a patient are comparable between caregivers for patients with either disease. A notable difference though, is that perceived caregiver strain for caregivers of AAA patients was highest 2 weeks after discharge, while the highest perceived caregiver strain was highest at discharge for caregivers of CRC patients (both medium effect sizes). This difference may best be explained by the combination of physical complaints and the psychological impact of the diagnosis of cancer, while an electively treated AAA often does not come with symptoms and may therefore have less impact on a caregivers’ mental burden. The timing of offering psychological support to caregivers should therefore be adjusted per diagnosis.

The strain of caregiver of octogenarians, patients that were dependent in ADL, cognitively impaired patients and of patients with a high burden of comorbidity was higher at baseline. In caregivers of these patients, the strain is not increased at discharge or 2 weeks post-discharge compared to baseline (with the exception of patients with a high burden of comorbidity at discharge), which suggests that the strain on these caregivers is affected more by these patient-related ‘risk’ factors, rather than the surgery itself. Caregivers of patients with these specific factors should therefore be better informed to prepare them for the upcoming care situation at home. Additionally, they may benefit from additional psychological support during the complete perioperative course, starting at the first outpatient clinic visit, prior to admission. Previous research demonstrated that psychological support, for example in prehabilitation programs starting prior to admission, is also recommended for patients [31]. Current prehabilitation programs may therefore combine psychological support for both patient and caregiver and add this as a component to the program [15].

In contrast, surgery significantly affects the strain of caregivers of 70–79-year olds, of patients who are independent in ADL, cognitively unimpaired patients and of patients with a lower burden of comorbidity at both discharge and 2 weeks post-discharge. For caregivers of patients without above-mentioned ‘risk’ factors, information provision and additional psychological support should focus on the early post-operative period specifically.

Open surgery and minimally invasive surgery affected the changes in caregiver strain over time differently. Health-care professionals should anticipate to this accordingly (i.e. additional psychological support for caregivers of patients who undergo open surgery may be desirable). It is advisable to offer this support to caregivers of patients with specific factors that may influence strain, as discussed above. In patients undergoing open AAA surgery, where perceived caregiver burden is over twice as high in the early postoperative period, this additional support may be specifically important.

The caregiver strain was higher in patients who were dependent in ADL at all time points, except at discharge. This finding is in line with previous studies which found that informal caregivers who provide assistance with activities of daily living had an increased risk for overload and with that, a higher caregiver burden [14, 32]. An earlier-mentioned study on caregiver strain in caregivers of patients with (orthopaedic) hip fracture surgery also mentioned pre-fracture functional status as an important factor to influence caregiver strain negatively after 1 year postoperatively [22].

The relation between caregiver burden and cognitive impairment has been extensively investigated in non-surgical patients in the past [33]. This study is one of the first to show a significant association between cognitive impairment of elderly surgical patients and perceived caregiver burden at baseline, discharge and after 6 months. Scores after 2 weeks and 12 months were also higher but did not reach statistical significance, possibly to the relatively low number of events. Two surgical studies have investigated this association before; one focussing on hip surgery patients and one on patients with intracranial tumours [34, 35]. In line with current research, the hip surgery study showed an association between both factors.

As mentioned earlier, preparing informal caregivers by providing them with information on the upcoming care situation at home and offering psychological support to caregivers during this period may help to lower the perceived burden. Another possible beneficial intervention may be to include them, if possible and wished, in caregiving during hospital stay, to prepare them for the upcoming care. They may be considered as partners in caring for older patients.

This research did not focus on characteristics of informal caregivers, even though these are also likely to influence the amount of strain experienced by caregivers. For example, comorbidities of the caregiver, being physically impaired and even gender are factors that may potentially influence a caregivers’ burden. Future research may therefore incorporate these factors in their study.

### Limitations

The CSI is a self-report questionnaire which focuses on the presence or absence of strain, experienced on different domains of informal caregiving. For each question, caregivers either experience strain, or they don’t. By extending the answer scale to 1 to 5, a better experience of strain may possibly be presented, making this test more accurate and reliable. A big advantage of the CSI however, is that it has a very good internal reliability (0.90), test-retest reliability (0.88) and a high level of internal consistency (α = .90) [36].

This study may have been underpowered to demonstrate significant effects of investigated factors on the caregiver strain in the informal caregivers of AAA patients. A trend can be observed in these analyses for AAA patients, however results often did not reach statistical significance.

This study is limited by using the combination of CRC and AAA, even though both conditions are fairly different. Including both can be justified by the fact that they have previously been used together in the definition of major abdominal surgery, are common in older patients, have a high rate of postoperative complications and require surgery as final treatment.

Another limitation is the relatively high percentage of attrition found in this study. At T3, only 61% of the informal caregivers filled in and returned the CSI questionnaire. Common reasons that were given were: “I don’t want to fill in the questionnaire, I’m not a patient.”, “This questionnaire does not apply to me or the patient.” and “This is a bad questionnaire.” Additionally, these types of questionnaires are limited by the relatively high risk of non-response bias. Caregivers of patients who require the most care are the ones most likely not to fill in follow-up questionnaires, especially when patients are considered too sick or weak to visit the outpatient clinic for their follow-up visits. The sensitivity-analysis that was performed supports this theory.

This non-response bias may also be an explanation for the lack of significant results when comparing the CSI of caregivers who provided care for patients who developed a delirium and patients that did not. Another explanation may be that the CSI is not sensitive for demonstrating the caregiver burden in patients with delirium. Future studies investigating the burden of caregivers of patients with delirium may therefore use questionnaires that were specifically designed for these patients [37].

## Conclusion

A not to be ignored burden is experienced by informal caregivers when providing care for elderly patients who undergo elective surgery for CRC and AAA, especially in the early postoperative period. The highest strain is experienced 2 weeks post-discharge when providing care for AAA patients and at discharge when providing care for CRC patients. After 1 year, the overall caregiver strain dropped below baseline. The burden on informal caregivers when providing informal care will increase in the upcoming years. These results emphasise the need for increased awareness for the impact of surgery on informal caregivers and the need for programmes to support these caregivers by preparing them for caregiving after discharge and to provide psychological support when necessary. This support should be timed according to the highest perceived strain per diagnosis.

Type of surgery is independently associated with the change in mean CSI scores over time; a bigger change in caregiver burden is observed after open surgery. The patient factors older age, dependency in activities of daily living, cognitive impairment and a higher burden of comorbidity are associated with a higher caregiver burden at baseline. Caregivers of patients with these factors may therefore benefit from information programmes and psychological support prior to surgery, possibly as a part of prehabilitation programs.

## Supplementary information

**Additional file 1: Appendix A.** Caregiver strain index in relation to patients’ age. **Appendix B.** Caregiver strain index in relation to patients’ dependency in ADL. **Appendix C.** Caregiver strain index in relation to type of surgery. **Appendix D.** Caregiver strain index in relation to patients’ burden of comorbidity. **Appendix E.** Caregiver strain index in relation to patients ‘cognitive status. **Appendix F.** Caregiver strain index in relation to development of delirium during admission.

## Data Availability

Data for this manuscript are part of a larger data file, which will be used for future publications. Data will therefore not be made publicly available.

## Abbreviations

CRC: Colorectal Carcinoma
AAA: Abdominal Aortic Aneurysm
CSI: Caregiver Strain Index
ADL: Activities of Daily Living
SNAQ: Short Nutritional Assessment Questionnaire
