# Supplementary material for: Caregiver strain on informal caregivers when providing care for older patients undergoing major abdominal surgery: a longitudinal prospective cohort study
Source: BMC Geriatr. 2020 May 19;20:178. doi: 10.1186/s12877-020-01579-8 (PMC7236465; doi:10.1186/s12877-020-01579-8)
Supplement: Supplementary file 1 — Additional file 1: Appendix A. Caregiver strain index in relation to patients’ age. Appendix B. Caregiver strain index in relation to patients’ dependency in ADL. Appendix C. Caregiver strain index in relation to type of surgery. Appendix D. Caregiver strain index in relation to patients’ burden of comorbidity. Appendix E. Caregiver strain index in relation to patients ‘cognitive status. Appendix F. Caregiver strain index in relation to development of delirium during admission. [file 12877_2020_1579_MOESM1_ESM.docx]

| **Appendix A. Caregiver strain index in relation to patients’ age** | | |  |
| --- | --- | --- | --- |
|  |  | |  |
|  | **70-79 years old** | **≥ 80 years old** | |
| **AAA** |  |  | |
| CSI T1, mean (SD) | 2.3 (2.1) N = 30 | 1.8 (2.0) N = 24 | |
| CSI T2, mean (SD) | 3.5 (3.0) N = 28 | 2.5 (2.8) N = 19 | |
| CSI T3, mean (SD) | 3.7 (3.5) N = 26 | 3.8 (3.5) N = 13 | |
| CSI T4, mean (SD) | 1.5 (1.9) N = 27 | 1.2 (1.8) N = 19 | |
| CSI T5, mean (SD) | 2.1 (3.0) N = 30 | 1.0 (1.6) N = 15 | |
|  |  |  | |
| **CRC** |  |  | |
| CSI T1, mean (SD) | 2.1 (2.6) N = 106 | 3.8 (3.5) N = 59^a^ | |
| CSI T2, mean (SD) | 3.0 (3.0) N = 82^b^ | 5.1 (3.8) N = 35^a^ | |
| CSI T3, mean (SD) | 2.6 (2.7) N = 79^b^ | 4.2 (3.9) N = 33^a^ | |
| CSI T4, mean (SD) | 2.0 (2.7) N = 93 | 2.9 (3.4) N = 43^b^ | |
| CSI T5, mean (SD) | 1.6 (2.5) N = 86 | 2.4 (3.2) N = 38^b^ | |
|  |  |  | |
| **All patients** |  |  | |
| CSI T1, mean (SD) | 2.2 (2.5) N = 136 | 3.2 (3.2) N = 83^a^ | |
| CSI T2, mean (SD) | 3.1 (3.0) N = 110^b^ | 4.2 (3.7) N = 54 | |
| CSI T3, mean (SD) | 2.9 (2.9) N = 105^b^ | 4.1 (3.8) N = 46 | |
| CSI T4, mean (SD) | 1.9 (2.5) N = 120 | 2.4 (3.1) N = 62^b^ | |
| CSI T5, mean (SD) | 1.7 (2.6) N = 116 | 2.0 (2.9) N = 53^b^ | |
|  |  |  | |
| a: Significant difference between groups for this time point  b: Significant difference in group between time point and outpatient clinic visit (T1) | | | |

| **Appendix B. Caregiver strain index in relation to patients’ physical status** | | |
| --- | --- | --- |
|  |  | |
|  | **KATZ-ADL = 6** | **KATZ-ADL ≤ 5** |
| **AAA** |  |  |
| CSI T1, mean (SD) | 2.1 (2.2) N = 42 | 1.9 (1.3) N = 12 |
| CSI T2, mean (SD) | 3.1 (3.1) N = 35 | 3.3 (2.4) N = 12 |
| CSI T3, mean (SD) | 3.5 (3.3) N = 29^b^ | 4.2 (4.0) N = 10 |
| CSI T4, mean (SD) | 1.2 (1.9) N = 36 | 2.0 (1.8) N = 10 |
| CSI T5, mean (SD) | 1.4 (2.4) N = 36 | 2.9 (3.3) N = 9^a^ |
|  |  |  |
| **CRC** |  |  |
| CSI T1, mean (SD) | 2.3 (2.7) N = 123 | 3.9 (3.6) N = 42^a^ |
| CSI T2, mean (SD) | 3.2 (3.2) N = 87^b^ | 4.7 (3.8) N = 30 |
| CSI T3, mean (SD) | 2.7 (2.7) N = 87 | 4.6 (4.1) N = 25^a^ |
| CSI T4, mean (SD) | 1.9 (2.4) N = 109 | 4.2 (3.9) N = 27^a^ |
| CSI T5, mean (SD) | 1.5 (2.3) N = 100 | 3.3 (3.7) N = 24^a^ |
|  |  |  |
| **All patients** |  |  |
| CSI T1, mean (SD) | 2.3 (2.6) N = 165 | 3.5 (3.4) N = 54^a^ |
| CSI T2, mean (SD) | 3.2 (3.2) N = 122^b^ | 4.3 (3.5) N = 42 |
| CSI T3, mean (SD) | 2.9 (2.9) N = 116^b^ | 4.5 (4.0) N = 35^a^ |
| CSI T4, mean (SD) | 1.7 (2.3) N = 145 | 3.6 (3.6) N = 37^a^ |
| CSI T5, mean (SD) | 1.5 (2.3) N = 136^b^ | 3.2 (3.6) N = 33^a^ |
|  |  |  |
| a: Significant difference between groups for this time point  b: Significant difference in group between time point and outpatient clinic visit (T1) | | |

| **Appendix C. Caregiver strain index in relation to type of surgery** | | |  |
| --- | --- | --- | --- |
|  |  | |  |
|  | **Minimally invasive** | **Open** | |
| **AAA** |  |  | |
| CSI T1, mean (SD) | 2.2 (2.2) N = 41 | 1.7 (1.2) N = 13 | |
| CSI T2, mean (SD) | 2.6 (2.5) N = 38 | 5.2 (3.8) N = 9^a, b^ | |
| CSI T3, mean (SD) | 2.9 (3.0) N = 31 | 6.6 (3.8) N = 8^a^ | |
| CSI T4, mean (SD) | 1.0 (1.5) N = 37^b^ | 2.9 (2.6) N = 9 | |
| CSI T5, mean (SD) | 1.8 (2.9) N = 36 | 1.4 (1.6) N = 9 | |
|  |  |  | |
| **CRC** |  |  | |
| CSI T1, mean (SD) | 2.9 (3.1) N = 146 | 1.8 (2.2) N = 19 | |
| CSI T2, mean (SD) | 3.6 (3.5) N = 102^b^ | 3.4 (3.2) N = 15 | |
| CSI T3, mean (SD) | 3.3 (3.3) N = 100^b^ | 1.7 (1.8) N = 12 | |
| CSI T4, mean (SD) | 2.4 (3.0) N = 120 | 1.9 (2.5) N = 16 | |
| CSI T5, mean (SD) | 1.9 (2.8) N = 114 | 0.5 (0.5) N = 10^b^ | |
|  |  |  | |
| **All patients** |  |  | |
| CSI T1, mean (SD) | 2.7 (2.9) N = 187 | 1.8 (1.9) N = 32 | |
| CSI T2, mean (SD) | 3.4 (3.3) N = 140^b^ | 4.1 (3.5) N = 24^b^ | |
| CSI T3, mean (SD) | 3.2 (3.2) N = 131^b^ | 3.7 (3.7) N = 20 | |
| CSI T4, mean (SD) | 2.0 (2.8) N = 157 | 2.3 (2.5) N = 25 | |
| CSI T5, mean (SD) | 1.9 (2.8) N = 150 | 1.0 (1.2) N = 19 | |
|  |  |  | |
| a: Significant difference between groups for this time point  b: Significant difference in group between time point and outpatient clinic visit (T1) | | | |

| **Appendix D. Caregiver strain index in relation to patients’ burden of comorbidity** | | |  |
| --- | --- | --- | --- |
|  |  | |  |
|  | **CCI < 7** | **CCI ≥ 7** | |
| **AAA** |  |  | |
| CSI T1, mean (SD) | 2.1 (2.1) N = 30 | 2.0 (2.0) N = 24 | |
| CSI T2, mean (SD) | 2.7 (3.1) N = 26 | 3.6 (2.7) N = 21^b^ | |
| CSI T3, mean (SD) | 3.4 (3.3) N = 20 | 4.1 (3.6) N = 19 | |
| CSI T4, mean (SD) | 0.9 (1.3) N = 29^b^ | 2.1 (2.5) N = 17 | |
| CSI T5, mean (SD) | 1.4 (2.2) N = 27 | 2.2 (3.2) N = 18 | |
|  |  |  | |
| **CRC** |  |  | |
| CSI T1, mean (SD) | 2.0 (2.3) N = 96 | 3.8 (3.5) N = 69^a^ | |
| CSI T2, mean (SD) | 2.9 (3.0) N = 72^b^ | 4.7 (3.8) N = 45^a^ | |
| CSI T3, mean (SD) | 2.7 (2.6) N = 68^b^ | 3.8 (3.9) N = 44 | |
| CSI T4, mean (SD) | 1.9 (2.4) N = 86 | 3.1 (3.6) N = 50 | |
| CSI T5, mean (SD) | 1.5 (2.3) N = 77 | 2.4 (3.2) N = 47^b^ | |
|  |  |  | |
| **All patients** |  |  | |
| CSI T1, mean (SD) | 2.0 (2.3) N = 126 | 3.3 (3.3) N = 93^a^ | |
| CSI T2, mean (SD) | 2.9 (3.0) N = 98^b^ | 4.3 (3.5) N = 66^a, b^ | |
| CSI T3, mean (SD) | 2.8 (2.7) N = 88^b^ | 3.8 (3.8) N = 63 | |
| CSI T4, mean (SD) | 1.6 (2.2) N = 115 | 2.8 (3.3) N = 67 | |
| CSI T5, mean (SD) | 1.5 (2.3) N = 104 | 2.3 (3.2) N = 65 | |
|  |  |  | |
| a: Significant difference between groups for this time point  b: Significant difference in group between time point and outpatient clinic visit (T1) | | | |

| **Appendix E. Caregiver strain index in relation to patients ‘ cognitive status** | | |
| --- | --- | --- |
|  |  | |
|  | **No cognitive impairment** | **Cognitive impairment** |
| **AAA** |  |  |
| CSI T1, mean (SD) | 2.1 (2.0) N = 53 | N/A |
| CSI T2, mean (SD) | 3.2 (2.9) N = 46^b^ | N/A |
| CSI T3, mean (SD) | 3.7 (3.4) N = 39^b^ | N/A |
| CSI T4, mean (SD) | 1.4 (1.9) N = 45 | N/A |
| CSI T5, mean (SD) | 1.8 (2.7) N = 44 | N/A |
|  |  |  |
| **CRC** |  |  |
| CSI T1, mean (SD) | 2.5 (2.8) N = 150 | 5.3 (3.8) N = 15^a^ |
| CSI T2, mean (SD) | 3.4 (3.3) N = 105^b^ | 5.8 (3.4) N = 12^a^ |
| CSI T3, mean (SD) | 2.9 (3.0) N = 104^b^ | 5.3 (5.0) N = 8 |
| CSI T4, mean (SD) | 2.1 (2.7) N = 123 | 4.7 (4.1) N = 13^a^ |
| CSI T5, mean (SD) | 1.6 (2.4) N = 113 | 3.8 (4.7) N = 11 |
|  |  |  |
| **All patients** |  |  |
| CSI T1, mean (SD) | 2.4 (2.6) N = 203 | 5.1 (3.8) N = 16^a^ |
| CSI T2, mean (SD) | 3.3 (3.2) N = 151^b^ | 5.3 (3.6) N = 13^a^ |
| CSI T3, mean (SD) | 3.1 (3.1) N = 143^b^ | 5.3 (5.0) N = 8 |
| CSI T4, mean (SD) | 1.9 (2.5) N = 168 | 4.4 (4.1) N = 14^a^ |
| CSI T5, mean (SD) | 1.7 (2.5) N = 157 | 3.5 (4.6) N = 12 |
|  |  |  |
| a: Significant difference between groups for this time point  b: Significant difference in group between time point and outpatient clinic visit (T1) | | |

| **Appendix F. Caregiver strain index in relation to development of delirium during admission** | | |
| --- | --- | --- |
|  |  | |
|  | **No delirium** | **Delirium** |
| **AAA** |  |  |
| CSI T1, mean (SD) | 2.1 (2.0) N = 52 | 2.0 (1.4) N = 2 |
| CSI T2, mean (SD) | 3.0 (2.9) N = 45 | 5.5 (3.5) N = 2 |
| CSI T3, mean (SD) | 3.6 (3.4) N = 37^b^ | 6.0 (4.2) N = 2 |
| CSI T4, mean (SD) | 1.4 (1.9) N = 44 | 1.5 (0.7) N = 2 |
| CSI T5, mean (SD) | 1.5 (2.3) N = 44 | N/A |
|  |  |  |
| **CRC** |  |  |
| CSI T1, mean (SD) | 2.7 (3.0) N = 150 | 3.5 (3.4) N = 15 |
| CSI T2, mean (SD) | 3.6 (3.5) N = 109^b^ | 3.8 (2.8) N = 8 |
| CSI T3, mean (SD) | 3.1 (3.2) N = 105^b^ | 2.9 (2.8) N = 7 |
| CSI T4, mean (SD) | 2.3 (2.9) N = 128 | 3.3 (3.9) N = 8 |
| CSI T5, mean (SD) | 1.8 (2.7) N = 116^b^ | 2.8 (2.8) N = 8 |
|  |  |  |
| **All patients** |  |  |
| CSI T1, mean (SD) | 2.5 (2.8) N = 202 | 3.3 (3.3) N = 17 |
| CSI T2, mean (SD) | 3.4 (3.3) N = 154^b^ | 4.1 (2.8) N = 10^b^ |
| CSI T3, mean (SD) | 3.2 (3.3) N = 142^b^ | 3.6 (3.2) N = 9^b^ |
| CSI T4, mean (SD) | 2.0 (2.7) N = 172 | 2.9 (3.5) N = 10 |
| CSI T5, mean (SD) | 1.7 (2.6) N = 160^b^ | 3.6 (3.5) N = 9 |
|  |  |  |
| a: Significant difference between groups for this time point  b: Significant difference in group between time point and outpatient clinic visit (T1) | | |
